# A case of C-PTSD: building trust and safety in the therapeutic relationship

**DOI:** 10.1192/j.eurpsy.2026.10267

**Published:** 2026-07-31

**Authors:** C. Tapoi

**Affiliations:** General Psychiatry, Alexandru Obregia Clinical Psychiatry Hospital, Bucharest, Romania

## Abstract

**Background:**

Complex Post-Traumatic Stress Disorder (C-PTSD), recognized in the ICD-11, is a mental disorder resulting from chronic and prolonged interpersonal trauma, such as physical or sexual abuse or neglect. In addition to the core symptoms of PTSD (re-experiencing, avoidance, and a persistent sense of threat), C-PTSD is characterized by disturbances in emotional regulation, a negative self-concept, and persistent difficulties in interpersonal interactions, including relationship avoidance and distrust of others. These features can be detrimental to the therapeutic relationship.

**Methods:**

We present the case of a 25-year-old woman who came to an outpatient psychiatric clinic with depressive mood, anhedonia, high levels of anxiety, insomnia, and pervasive feelings of shame and worthlessness. Her history included physical abuse within her family of origin and prolonged bullying during childhood and adolescence. She was diagnosed with C-PTSD and major depressive disorder and was started on antidepressant medication and referred for psychotherapy.

**Results:**

During the first nine months of treatment, the patient showed improvement in depressive mood; however, feelings of worthlessness, marked anxiety, and hyperarousal persisted. She subsequently missed two consecutive monthly appointments. At the following visit, she disclosed having recently exited an abusive intimate relationship. She reported feeling too ashamed to discuss the situation earlier but decided to leave her boyfriend after experiencing physical assault. At that time, she presented with panic attacks, emotional lability, flashbacks, hyperarousal, insomnia. Pharmacological treatment was resumed, and psychotherapy was re-initiated.

**Discussion:**

Leaving the abusive relationship and finding the capacity to disclose her experience and ask for help represented a significant therapeutic turning point. The gradual establishment of trust and perceived safety within the therapeutic relationship enabled the patient to address ongoing trauma-related difficulties.

**Conclusion:**

C-PTSD is a condition associated with high vulnerability, which requires a careful approach. Patients with C-PTSD often struggle with trust and may delay disclosure of ongoing abuse. Providing a consistent sense of safety, emotional attunement, and non-intrusive support is essential to fostering engagement and facilitating recovery.

**Disclosure of Interest:**

None Declared

